# Monkeypox: what do dental professionals need to know?

**DOI:** 10.1038/s41415-022-5079-8

**Published:** 2022-10-14

**Authors:** Charifa Zemouri, Edgar O. Beltrán, Richard Holliday, Nicholas S. Jakubovics, James R. Allison

**Affiliations:** 4141545028001 Zemouri Public Health Research and Consultancy, Amsterdam, The Netherlands; 4141545028002 UNICA – Caries Research Unit, Research Department, Universidad El Bosque, Bogotá, Colombia; 4141545028003 School of Dental Sciences, Faculty of Medical Sciences, Newcastle University, Newcastle upon Tyne, UK; Newcastle upon Tyne Hospitals NHS Foundation Trust, Newcastle upon Tyne, UK; 4141545028004 School of Dental Sciences, Faculty of Medical Sciences, Newcastle University, Newcastle upon Tyne, UK

## Abstract

Infection control is critical for the safe delivery of dental care. Infection control practices must be responsive to emerging and re-emerging infectious diseases and outbreaks, as was clearly seen during the peak of the COVID-19 pandemic. An emerging global outbreak of the monkeypox virus has again raised potential challenges for infection control in dentistry. Monkeypox is an infectious disease, characterised by a rash affecting the skin and soft tissues, including the oral cavity. Previously, cases were mostly seen following contact with infected animals in Central and West Africa, with limited human-to-human transmission within and outside of these areas. However, since May 2022, sustained human-to-human transmission has occurred globally. Monkeypox can be transmitted via close contact with an infected person, contaminated objects and surfaces, or by droplets and possibly aerosols, which is therefore of potential importance to dental settings. This article discusses the relevance of monkeypox to dental professionals, the typical presentation of the disease, its potential impact on infection prevention and control practices and the delivery of dental services. The current monkeypox outbreak highlights the need for a more sustained programme of research into dental infection control that can provide a solid evidence base to underpin preparedness planning for future outbreaks and pandemics.

## Key points

- Monkeypox is a re-emerging viral infectious disease that may have relevance to dental professionals due to the risk of cross-infection or occupational exposure.
- The characteristic rash seen in monkeypox is often widespread but commonly affects the oral cavity and perioral structures, and cervical lymphadenopathy is a common feature.
- Usual infection prevention and control precautions are likely to be effective to control the risk posed by monkeypox in dental settings. However, respiratory protection is required when treating confirmed or probable cases.
- Further research is important to understand the risks from emerging and re-emerging viral infectious diseases in dental settings and how to control these risks.

## Introduction

Monkeypox is a re-emerging zoonotic infectious disease caused by the monkeypox virus (MPXV) which is a member of the Orthopoxvirus genus in the family *Poxviridae*. MPXV is a Hazard Group 3 pathogen and can only be handled in specialist facilities.^1^ Other members of this genus include the variola virus which causes smallpox and the related vaccinia virus on which the modern smallpox vaccine is based. Small rodents and other mammals, endemic to Central Africa, are thought to be the natural reservoirs of MPXV.^2^ However, the virus' nomenclature derives from the first described pox-like disease in captive monkeys in 1958.^3^ MPXV has historically been classified into two genetically distinct groups, or clades, named after the geographic location of first identification.^4^ In this paper, we follow the recently proposed non-discriminatory and non-stigmatising classification described by Happi *et al.*^5^ who proposed the terms Clade 1 to describe that previously known as the Congo Basin clade and Clades 2 and 3 which together comprise the previously termed West African clade. Clade 3 includes genome sequences isolated from individuals from the UK, Israel, Nigeria, USA and Singapore between 2017-2019, as well as from the 2022 outbreak. The placeholder name 'hMPXV' has been proposed to distinguish this clade from others. The World Health Organisation (WHO), in collaboration with the International Committee on Taxonomy of Viruses and the international scientific community may have recommended changes to the taxonomy of MPXV or the nomenclature of the virus, its clades and variants, and the disease it causes since submission of this paper. The terms used in this article were current at the time of submission.

**Fig. 1:**
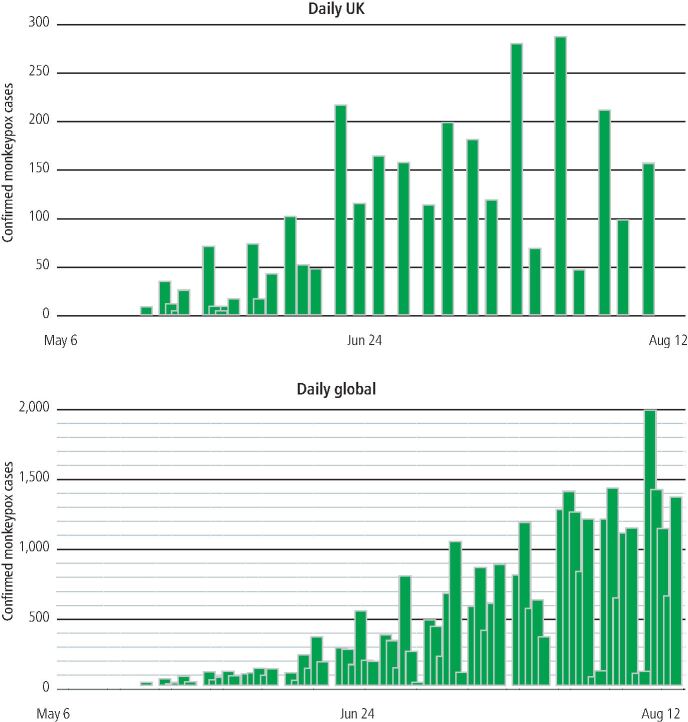
Daily monkeypox cases in 2022 by date of test report. Upper panel shows UK data, lower panel shows global data. Data obtained from Mathieu *et al*.^15^

The first case of monkeypox in humans was described in 1970^6^ and the disease is currently endemic in Central and West African countries, such as Cameroon, Nigeria and the Democratic Republic of the Congo.^7^^,^^8^^,^^9^ In the past, sporadic MPXV infections, related to travel from areas where the disease is endemic, occurred outside of the African continent.^10^ Monkeypox was designated an airborne 'high consequence infectious disease' by the UK Health Security Agency (UKHSA; then Public Health England) in 2018^11^ and subsequent disease surveillance between 2018-2021 identified seven cases of monkeypox in the UK, of which, three were attributed to onward transmission within the UK.^12^ On 6 May 2022, the UKHSA reported a case of monkeypox in the UK and multiple cases were identified shortly after.^13^^,^^14^ Confirmed cases have continued to rise and as of 11 August 2022, there were 3,023 confirmed cases of monkeypox in the UK (Fig. 1).^15^ A large proportion of cases were known to be residents of London (71%)^16^ and almost all were men (98.8%), with the majority identifying as gay, bisexual, or men who have sex with men (GBMSM).^13^ Globally, as of 15 August 2022, a total of 35,874 confirmed monkeypox cases had been reported in 89 countries, of which, 82 countries had not reported monkeypox cases before the current outbreak.^15^^,^^17^ As of 23 July 2022, the WHO declared the monkeypox outbreak a public health emergency of international concern.^18^

## Routes of transmission

Zoonotic (animal-to-human) transmission of MPXV occurs from infected animals via bites, scratches and close contact.^7^ Contact with infected captive animals or their cages and bedding may also lead to the transmission of monkeypox.^19^^,^^20^^,^^21^ The 2022 outbreak is characterised by human-to-human transmission and sexual contact has been documented in 91.7% of cases.^22^ Nevertheless, it is not yet clear whether the virus spreads directly through sexual contact or whether transmission is driven by close contact with an infected person during sexual activity, for example, as a result of direct contact with skin lesions or via bodily fluids, such as saliva and respiratory secretions.^9^^,^^23^ The latter may occur via droplets, as well as by contamination of surfaces^7^^,^^12^ and transmission via infectious aerosols cannot be excluded.^24^^,^^25^ The main sites of MPXV entry are the mucous membranes, non-intact skin, open wounds and by inhalation.^26^

## Clinical presentation of monkeypox

Monkeypox has a relatively long incubation period which can range from 5-21 days. The disease is characterised by two distinct phases.^9^^,^^12^ Symptoms begin with an initial prodromal illness which comprises:

- Fever
- Lymphadenopathy Myalgia
- Fatigue
- Headache
- Back pain.

**Fig. 2:**
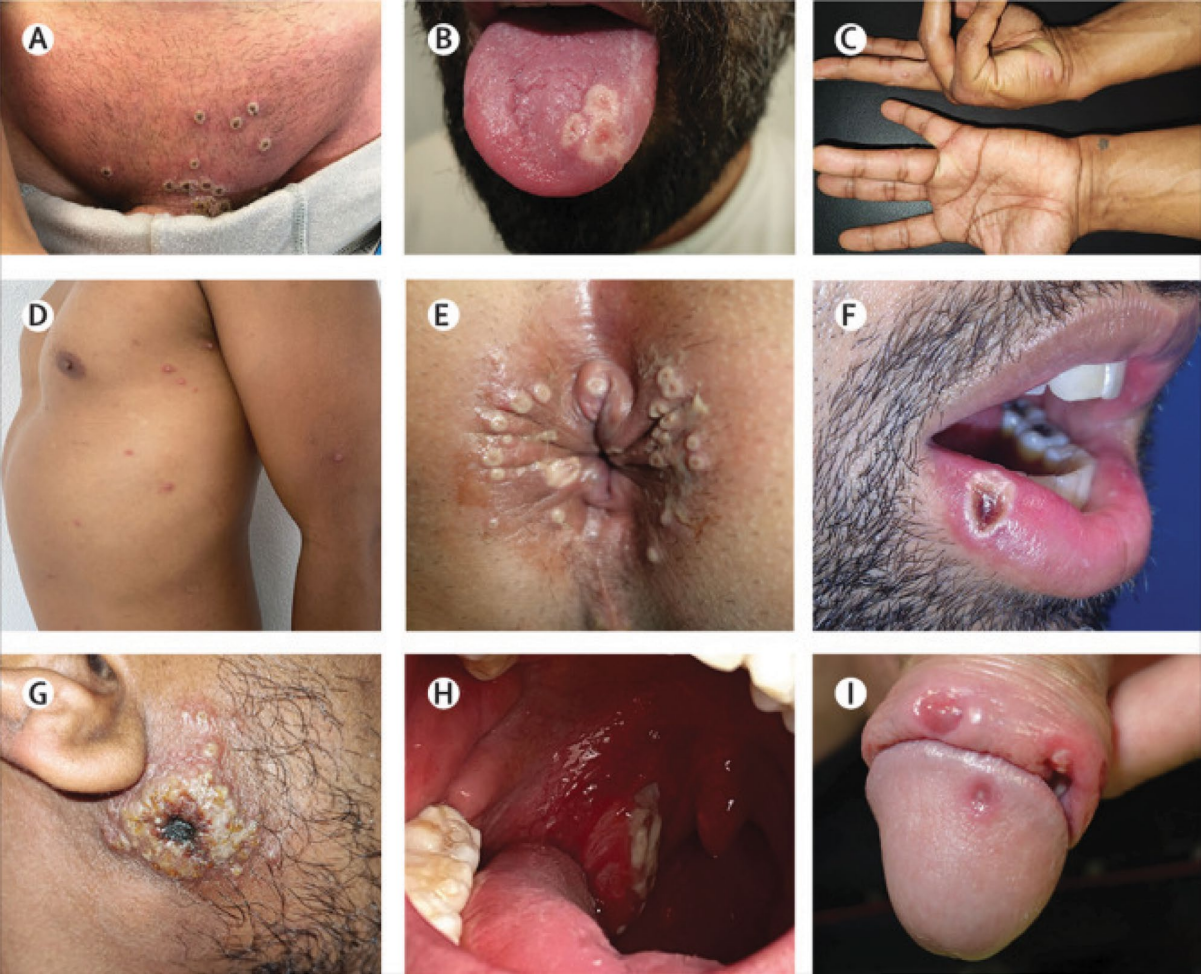
Lesions in patients with monkeypox. a) Pubic lesions showing umbilication with a central crust. b) Confluent ulcerated lesions affecting to tongue dorsum. c) Vesicular lesions affecting the palms and fingers. d) Papules and pustules affecting the trunk and arms. e) Umbilicated pustules affecting the anus. f) Ulcerated lesion with raised margins affecting the mucosa of the lower lip. g) Large, crusted lesion affecting the site of primary inoculation on the skin of the cheek. h) Enlarged and erythematous tonsil with ulceration and fibrinous slough. i) Ulcerated lesions affecting the glans and foreskin of the penis. Reprinted from *The Lancet*, Tarín-Vicente *et al*., 'Clinical presentation and virological assessment of confirmed human monkeypox virus cases in Spain: a prospective observational cohort study', Copyright 2022, with permission from Elsevier^45^

The initial illness is followed usually within a few days of the onset of initial symptoms by a rash. Individual lesions progress through the following appearances (Fig. 2):

- Macules (flat-based lesions)
- Papules (raised firm lesions)
- Vesicles (lesions with clear fluid)
- Pustules (lesions with yellowish fluid)
- Crusted lesions.

Monkeypox has a spectrum of clinical manifestations. In approximately 70% of monkeypox cases, the oral mucosa is affected and lesions can also affect the pharynx.^9^^,^^27^^,^^28^ Anogenital lesions and rectal pain have also been commonly reported during the current outbreak and as the outbreak advances, further clinical symptoms may be added to the case definition.^29^^,^^30^ The lesions may be distributed peripherally or might affect the entire body during severe illness^31^ and may number from several lesions to thousands. The extremities, genitalia and face are most commonly affected sites and although most cases are self-limiting, complications can include pneumonitis, encephalitis and secondary bacterial infections.^12^ Patients are considered to be no longer infective when the crusts have fallen off and new skin or mucosa is developed, which can take up to four weeks.^12^^,^^31^^,^^32^

## Risk to healthcare workers

Given the mode of monkeypox transmission, healthcare workers may be at increased risk of acquiring the infection through close and extended contact with infected patients.^12^^,^^23^^,^^33^ Where the prevalence of monkeypox is low, clearly the probability of a healthcare worker encountering an infective patient is low; however, in populations where community transmission is high, this risk may be increased. As some authors have highlighted,^34^^,^^35^ dental practitioners may be at additional risk due to the production of droplets and aerosols during dental procedures and prolonged close contact with patients.^36^ Fluid from skin or oral lesions containing MPXV, or from blood and saliva, might become dispersed into the environment via droplets and aerosols, or by direct contact with patients, producing a risk of occupational exposure to dental professionals and nosocomial infection to other patients.

Although the airborne route is not likely to be the primary mode of monkeypox transmission, droplet transmission is a significant route and aerosolisation during aerosol-generating procedures (AGPs) is possible.^37^ MPXV has been shown to remain infective in aerosols for several hours^24^ and infection via aerosolised MPXV has been demonstrated in animal models.^38^ As with other infectious agents, the risk of airborne transmission depends on: the duration of exposure; infectious dose; presence of respiratory protective equipment; and environmental parameters, such as humidity, temperature and ventilation.^39^ Therefore, dental practitioners must remain vigilant to the risk of treating patients with monkeypox, particularly in areas where community transmission is high.

## Awareness and management of a possible or probable case

In general, monkeypox is likely to cause little risk to dental professionals, as although the current outbreak is clearly of great importance to public health, the number of cases is low compared to the millions of patients seen by NHS dentists in the UK every year.^40^ Despite this, a large proportion of the population use dental services and so during periods of increased community transmission, it is possible that some patients with monkeypox may seek dental care. It is therefore important that dental professionals have an understanding of the disease and its clinical presentation. Patients who present with an unexplained rash on any part of their body and one or more symptoms typical of monkeypox should prompt dental professionals to consider MPXV as a possible cause. In line with UKHSA guidance, monkeypox is a probable diagnosis in patients with a presentation consistent with monkeypox, who have also: i) been in contact with a person with probable or confirmed monkeypox in the 21 days before symptom onset; ii) travelled to West or Central Africa in the 21 days before onset of symptoms; or iii) are GBMSM.^41^ In the case of suspected monkeypox, the patient should be provided with a surgical mask and asked to return home to isolate and await further advice. The dental professional should then contact their local health protection team for guidance.

## Infection prevention and control measures

Although it is unlikely that dental professionals would encounter a patient with monkeypox in the dental setting, either knowingly or unknowingly, the main transmission risk would likely be from direct contact with skin lesions or clothing that has been in contact with lesions. As such, standard infection prevention and control (IPC) precautions recommended in the *National IPC manual for England*,^37^ such as the use of gloves, aprons, fluid-resistant surgical masks (FRSM) and eye protection where appropriate, would provide protection from contact transmission. There is not yet sufficient data to confirm or refute airborne transmission as a major route of transmission. However, given that droplet transmission is known to occur, AGPs, such as the use of high-speed handpieces and ultrasonic instruments, present an elevated risk of transmission. Standard IPC precautions of a FRSM and eye protection during AGPs would be below the recommended level of protection for conducting an AGP in a patient with monkeypox. Instead, respiratory protection, such as a filtering face piece 3 (FFP3) mask, fluid-resistant gown and visor would be required. It should be noted that where community prevalence remains low, this scenario is improbable.

Any elective dental treatment in patients with possible, probable, or confirmed monkeypox should be delayed until monkeypox is excluded or the patient is no longer infective. In the unlikely event that such a patient required emergency dental treatment, which was not possible to defer, AGPs should be avoided wherever possible. UKHSA guidance for ambulatory settings recommends that patients should be placed in an individual room rather than a shared waiting area and should be given an FRSM to wear to reduce droplet transmission risk. Current advice is that pregnant women and severely immunocompromised individuals should not provide care for such patients.^25^ Appropriate PPE when caring for such a patient would be an FFP3 respirator, fluid-resistant gown, gloves and eye protection.

## Vaccination

Since the eradication of smallpox in 1980 by widespread vaccination, MPXV has become the most important member of the *Orthopoxvirus* genus in terms of relevance to human health and disease.^7^ Because the two viruses are closely related, smallpox vaccination offers a degree of protection against monkeypox.^10^^,^^42^ However, cessation of smallpox vaccination since eradication of the disease will likely have reduced population immunity to Orthopox viruses, such as MPXV. A third-generation smallpox vaccine (Modified Vaccinia Ankara - Bavarian Nordic [MVA-BN]), which uses an attenuated vaccinia virus, is marketed under the name JYNNEOS in the USA and is licenced for the prevention of smallpox and monkeypox. The same vaccine, marketed under the name Imvanex, is licenced in the UK for the prevention of smallpox; the use of the MVA-BN vaccine for monkeypox prevention in the UK is, however, an off-label use.^43^ Vaccination in the UK is currently considered for pre-exposure prophylaxis in high-risk groups and for healthcare and laboratory workers likely to be exposed to patients with monkeypox or work with MPXV samples. The vaccine is provided as post-exposure prophylaxis for the contacts of confirmed cases.

## Primary presentation to the dental clinic and differential diagnosis

**Fig. 3:**
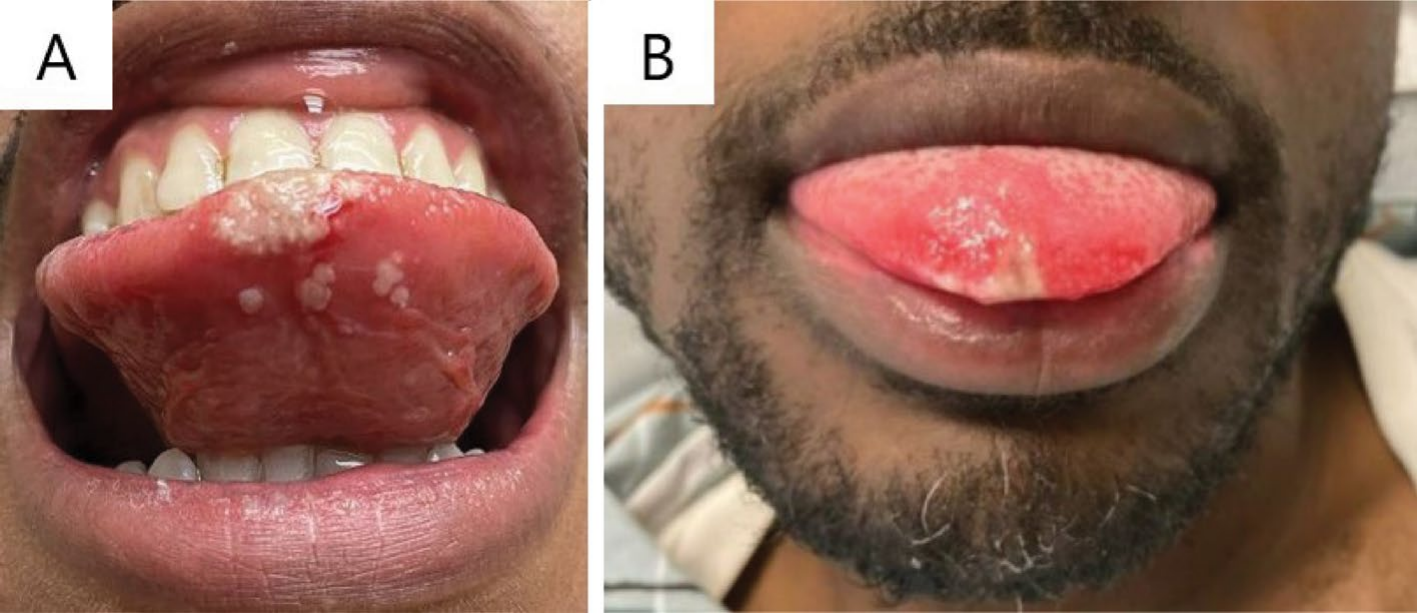
Oral monkeypox lesions. a) Confluent vesicular and ulcerated lesions affecting the tip and ventral surface of the tongue. b) Ulcerated lesion affecting the tongue tip with surrounding erythema. The lesion had been present for three days before the patient's presentation. Reprinted from *Journal of Oral and Maxillofacial Surgery*, Peters *et al*., 'Oral manifestations of monkeypox: a report of two cases', Copyright 2022, with permission from Elsevier^44^

Although acutely unwell patients, or those with a widespread, pox-like rash, are likely to present to medical colleagues, it is possible that patients with more limited disease affecting the head and neck might first present to dental professionals. For example, a common symptom of monkeypox is lymphadenopathy, which may affect the cervical lymph nodes; similarly, patients with a limited rash may have lesions of the oral cavity or perioral region only (Figures 2 and 3).^44^^,^^45^ Oral and perioral monkeypox lesions may present as round, pox-like lesions around the lips, chin and nose. In the oral mucosa, ulcerated lesions may commonly be found on the tongue, the buccal mucosa and on the tonsils.^29^ The latter may be easily mistaken for other infections, such as tonsillitis. Lesions affecting the oral mucosa may not progress in the characteristic manner of cutaneous monkeypox lesions, adding further complexity to the differential diagnosis. As above, where monkeypox is possible or probable, further questions should be asked to identify risk factors and advice sought from the local health protection team.

A differential diagnosis may include lesions caused by varicella-zoster virus, including chickenpox and herpes zoster (shingles), although the itchy maculopapular lesions of chickenpox are unlikely to be umbilicated as monkeypox lesions often are and herpes zoster presents with a typically dermatomal distribution of numerous vesicles which coalesce and crust. Molluscum contagiosum, a condition caused by the molluscum contagiosum virus, which is another member of the family *Poxviridae*, may cause a similar appearance, with raised, pink lesions with a central dimple. In cases where oral ulceration is an early presenting symptom,^44^ other causes, such as traumatic ulceration, should be considered, although concomitant systemic symptoms, such as fever and lymphadenopathy, would make an infectious cause more likely.

At present, it is highly unlikely that dental professionals will encounter patients with possible, probable, or confirmed monkeypox; however, practitioners in geographic areas with clusters of cases or those who serve communities that are particularly affected may be more likely to see patients with monkeypox. Additionally, it is possible that the primary presentation of monkeypox could be to a dental professional in cases where the disease is mild and limited to the head and neck or with oral or perioral lesions. Of course, the probability of encountering a case may change if community prevalence continues to increase, but whatever the fate of the current epidemic, dental professionals should be mindful of the possibility of encountering patients with monkeypox and should consider how their team might manage such an eventuality.

## Future implications for dental healthcare

Dentistry has a long history of changing IPC practices in light of new or changing diseases.^46^ One of the greatest challenges to dental infection control was posed by the emergence of the human immunodeficiency virus (HIV) in the 1980s.^47^ The current monkeypox outbreak has some similarities to the emergence of HIV, including that it is primarily driven by sexual contact between men. As such, patients may be reluctant to disclose a diagnosis due to perceived stigma associated with the disease. In contrast to HIV, it appears that monkeypox infection resolves in around one month and individuals are no longer infectious beyond this point. Therefore, risks of transmitting monkeypox during healthcare procedures would only occur during the prodromal or acute phases of the infection, which dramatically reduces the likelihood of dentists coming into contact with infective cases.

General awareness of the importance of IPC has been heightened following the COVID-19 pandemic. Dental professionals are now familiar with terms such as 'AGP', 'FFP3' and 'LFT' (lateral flow test), which previously would have been the preserve of IPC experts only. Although the current monkeypox outbreak does not currently pose such high risks to public health and the delivery of dental care, it re-emphasises the need to be alert to new or re-emerging infectious diseases. Nevertheless, high-quality evidence on the risk of transmission of viruses in dental settings and during dental procedures, as well as how to control this risk, remains sparse^36^^,^^48^ and this is also true of healthcare more widely.^49^ It is vitally important that dentistry, wider healthcare and research funding bodies continue to prioritise research into IPC to ensure that services remain resilient in future emerging and re-emerging infectious disease outbreaks. We call upon professionals to follow current guidance and existing recommendations. Furthermore, it may be that current IPC guidelines require updating to ensure resilience in the face of future viral disease outbreaks.

## Conclusion

Monkeypox is transmitted through direct contact and respiratory droplets, although airborne transmission cannot be excluded and would remain a risk during AGPs. Usual IPC precautions would likely reduce the risk of transmission from an infected person in the dental setting; however, respiratory protection would be necessary during AGPs. An awareness of the typical presentation of monkeypox is important for dental practitioners in the unlikely event that an infected patient presents to them. The monkeypox outbreak has once again highlighted an urgent need for strong evidence to underpin IPC measures to protect against current and emerging infectious diseases. Continued research is necessary to ensure we are well-prepared for future challenges.
